# Identifying homogeneous subgroups of patients and important features: a topological machine learning approach

**DOI:** 10.1186/s12859-021-04360-9

**Published:** 2021-09-20

**Authors:** Ewan Carr, Mathieu Carrière, Bertrand Michel, Frédéric Chazal, Raquel Iniesta

**Affiliations:** 1 Department of Biostatistics and Health Informatics, Institute of Psychiatry, Psychology and Neuroscience, King’s College London, London, UK; 2 Inria Sophia-Antipolis, DataShape Team, Biot, France; 3 Ecole Centrale de Nantes, LMJL – UMR CNRS 6629, Nantes, France; 4 Inria Saclay, Ile-de-France, Alan Turing Building, Palaiseau, France

**Keywords:** Topological data analysis, Clustering, Machine learning

## Abstract

**Background:**

This paper exploits recent developments in topological data analysis to present a pipeline for clustering based on Mapper, an algorithm that reduces complex data into a one-dimensional graph.

**Results:**

We present a pipeline to identify and summarise clusters based on statistically significant topological features from a point cloud using Mapper.

**Conclusions:**

Key strengths of this pipeline include the integration of prior knowledge to inform the clustering process and the selection of optimal clusters; the use of the bootstrap to restrict the search to robust topological features; the use of machine learning to inspect clusters; and the ability to incorporate mixed data types. Our pipeline can be downloaded under the GNU GPLv3 license at https://github.com/kcl-bhi/mapper-pipeline.

## Background

Past studies have demonstrated the importance of studying heterogeneity in treatment response or prognostic outcome [1]. Differing rates or trajectories of response may be associated with relevant clinical and biological characteristics of interest and identifying subgroups of patients with shared outcomes may allow treatments to be targeted more effectively. Recent studies, including COVID-19 research, have highlighted the need for clustering algorithms for mixed data types [2, 3]. This paper presents a novel pipeline for clustering using topological data analysis (TDA) that brings several advantages over existing approaches. These include the ability to identify homogeneous clusters with respect to an outcome of interest; to incorporate prior knowledge into the clustering process; and the use of machine learning to examine the composition of derived clusters.

TDA is a growing field (see Additional file 1: Table S2) providing tools to infer, analyse, and exploit the shape of data [4, 5]. TDA has seen increasing adoption in recent years [6]. It holds particular promise as a set of tools to further precision medicine [7, 8] where we often want to identify groups of patients with similar treatment or prognostic outcome. Our pipeline focuses on Mapper [9], a clustering algorithm to identify topological features in complex data that has shown big potential in uncovering homogeneous subgroups sharing common characteristics [10].

### The Mapper algorithm for clustering

The Mapper algorithm [9] reduces complex data into a one-dimensional graph. It assumes a finite *point cloud* for which distances between any two points can be computed; and a *filter* function that assigns a value to each point in the dataset. Shown in Additional file 1: Fig. S1, the algorithm then: (i) divides the range of the filter function into a number of smaller overlapping intervals; (ii) finds the sets of data points whose values assigned by the filter function lie within each interval; (iii) decomposes each set into clusters using a chosen clustering algorithm (e.g. DBSCAN); and (iv) represents each cluster as a node. Nodes are connected by an edge if the clusters intersect non-trivially (i.e. they share a minimum number of individuals). The connected nodes thus form shapes. Typical shapes that appear in the graph are ‘loops’ (continuous circular segments) and ‘flares’ (long linear segments) [11].

The above procedure requires the user to define several parameters, such as the number of intervals and their percentage of overlap, the choice of clustering algorithm, and the threshold at which to connect nodes. Although Mapper is independent of the choice of clustering algorithm, it is common practice to use clustering methods with parameters that can be derived automatically (e.g. hierarchical clustering or DBSCAN). Users are also recommended to compare several clustering techniques, particularly when analysing small sample sizes [12]. Of particular relevance here is the choice of *filter*—a lens through which to view the point cloud. Changing the filter will result in a different ordering of values along the range of the filter, different overlapping intervals, and produce differing clusters, nodes and features.

Mapper offers several advantages over traditional clustering algorithms. Of particular relevance here is the integration of prior knowledge through the selection of the filter. By choosing one or more variables as a lens through which to view their data, users can orient the clustering algorithm towards theoretically relevant markers. Another advantage of Mapper is that it allows identification of subgroups belonging to ‘flares’ and ‘loops’, rather than the ‘connected components’ detected in classical clustering algorithms. By forming clusters within overlapping intervals of the data it avoids artificially breaking continuous variation into discrete clusters. Mapper can also be used as a form of feature selection, by including all variables as filters and evaluating their ability to discriminate subpopulations of interest.

## Implementation

Our pipeline performs a grid search of the parameter space by evaluating all combinations of input parameters. It proceeds in five stages:

1. Compute the Gower distance matrix [13] for the input dataset;
2. Enumerate all combinations of input parameters;
3. For each set of input parameters, (i) compute Mapper graph; and (ii) identify statistically significant representative topological features (i.e. Clusters);
4. Among all mapper graphs, rank clusters in terms of their impurity [14, p. 309] with respect to a chosen outcome or variable of interest; and
5. Visualise and summarise the top five clusters.

In an example application with a sample of 430 patients, we aimed to identify subgroups that were similar in terms of baseline clinical and genetic characteristics (140 variables) as well as outcome (remission following 12 weeks of treatment). Input data were a mixture of categorical and continuous variables.

### Step 1

We first construct a distance matrix for clinical and genetic predictors from the input dataset. To allow a mix of continuous and categorical variables we use the Gower distance [13] implemented in the gower package for Python [15]. This computes distances between pairs of variables using appropriate measures (Manhattan distance metric for continuous variables; Sørensen-Dice coefficient for categorical) and then combines these into a single distance averaged over all variables ranging from 0 to 1. Importantly, outcomes are not used to derive the Gower matrix.

### Step 2

We then define sets of input parameters for the Mapper algorithm. While some parameters can be derived automatically [16] several must be specified by the user including: (i) the choice of filter(s); (ii) gain; (iii) resolution; and (iv) clustering algorithm. ‘Gain’ and ‘resolution’ control how the range of the filter function is divided into intervals (see Additional file 1: Fig. S1). The ‘gain’ refers to the overlap between consecutive intervals whereas the ‘resolution’ refers to the diameter of the intervals. By choosing the number of intervals and the percentage overlap between them, the user can adjust the level of the detail at which to view their data. For a single filter, resolution can be derived automatically, but must be specified when combining multiple filters. We enumerate all combinations of parameters and store these as inputs for subsequent steps (i.e. a grid search). Since optimal parameters will depend on the input dataset, we recommend exploring a range of values. Our example application considered combinations of:

i. Five *filters* comprising two ‘data filters’ based on continuous predictor variables; two ‘computed filters’, based on the first two components from Principal Components Analysis (PCA); and combinations of data and computed filters.
ii. Four values for *gain* (0.1, 0.2, 0.3, 0.4);
iii. Six values for *resolution* (1, 3, 5, 10, 30, 50);
iv. Two clustering algorithms (Density-based spatial clustering of applications with noise, DBSCAN; and Agglomerative Clustering).

In our application the ‘data filters’ were theoretically chosen. We considered as filters variables known to be important for the outcomes in question. However, an alternative approach could be to consider all continuous variables in the input dataset as candidate filters. Following the steps described below, the pipeline would then identify the ‘optimal’ clusters having considered all candidate filters. This approach would be computationally intensive since the search grid would expand substantially. However, by allowing all filters to considered and ranked (based on clusters homogeneity in terms of the outcome variable, as described below) this process would provide an effective form of feature selection; the ranked list of filters would indicate their importance.

### Step 3

For each set of input parameters, we (i) compute the Mapper graph; and (ii) identify representative topological features; and (iii) evaluate the statistical significance of each representative feature with the bootstrap. This uses re-sampling methods to assess whether a given topological feature is robust to small variations in the dataset [16].

### Step 4

From the list of candidate topological features, we rank clusters based on the best separation with regards to the chosen outcome of interest (i.e. homogeneity within cluster). We first exclude non-significant or small features ($<5$ or $>95$ percent of sample). We then calculate homogeneity for each feature with respect to the chosen outcome of interest as well as percentage improvement in homogeneity compared to overall homogeneity of the sample. For binary outcomes, homogeneity is assessed using Gini impurity [14, p. 309] defined as $1 - (1 - p)^2 - p^2$ where *p* is the proportion of individuals in the feature experiencing the outcome of interest. Lower values indicate lower Gini impurity, down to a minimum of 0 at which point all individuals in the cluster fall into a single outcome category. For continuous outcomes homogeneity could be measured using the standard deviation. This is calculated for each candidate feature separately as well as for the overall sample. Finally, we sort all features by their percentage improvement in homogeneity.

### Step 5

We select the top five features and describe each by:

- Describing differences in each predictor between members and non-members of the chosen feature, including *p*-values to indicate statistical significance;
- Predicting membership to the feature using gradient boosted trees (*XGBoost*).
- Visualising the Mapper graph and highlighting the chosen topological feature.

## Results

We applied our pipeline to the GENDEP dataset [17], described in Additional files. We compared the performance obtained with our pipeline to that obtained with k-means clustering. We assessed impurity for a categorical outcome (remission at 12 weeks; 1 = ‘Yes’, 0 = ‘No’) using each method. Presented in Additional files, we found that the top five clusters from our pipeline outperformed the five cluster solution from k-means clustering in terms of outcome impurity (Gini for our clusters 0.30–0.38; for k-means 0.33–0.50). Clusters from our method also showed the highest reduction in impurity when compared to the whole sample. Overall, we found that our pipeline outperformed k-means in identifying homogeneous clusters in terms of an external outcome distribution. These results are shown in Additional file 1: Table S1. The composition of the five best-performing clusters from our pipeline is presented in Additional file 2: Table S3. A summary of the software used in our pipeline can also be found in Additional files.

## Discussion

While several software implementations of the Mapper algorithm exist—including open source packages such as *Python Mapper* and *KeplerMapper* and proprietary software such as *Ayasadi*—our pipeline allows identification of homogeneous subgroups with respect to one or more variables of interest. Mapper requires users to specify several parameters such as the ‘gain’ (the overlap between consecutive intervals) and ‘resolution’ (the diameter of the intervals). In contrast to trial-and-error used in most existing applications, our pipeline enables exploration of this parameter space to be informed by a chosen predictor or outcome of interest. By ranking clusters by homogeneity with respect to a chosen outcome or predictor, we automatically derive optimal tuning parameters. Secondly, whereas researchers typically inspect the derived clusters by comparing relevant variables one-by-one across clusters, we use machine learning to examine clusters from a predictive and multivariable perspective. Thirdly, we use the bootstrap to exclude statistically insignificant topological features thereby focusing our inferences on clusters that are robust to outliers and inferable to the population [16]. Fourth, we allow mixed data types via the Gower distance matrix [13]. Fifth, we identify ‘representative’ topological features and display these visually. While software exists to visualise the Mapper graph, most implementations emphasise membership to individual nodes rather than topological features.

Applied to a particular dataset our pipeline outperformed k-means in identifying homogeneous clusters of patients with respect of an outcome variable. The performance of the Mapper algorithm has been previously compared against standard algorithms in other datasets, including hierarchical clustering, k-means, DBSCAN, Single-linkage and Complete and average linkage clustering [18, 19]. In general, methods produced similar clusters when data points in the sample were vastly different, but Mapper was found to be more sensitive to small variations. Methods that do not require the number of clusters to be pre-specified (such as Mapper) showed a key limitation related to density issues. When clusters with different densities were present, Mapper tended to select only clusters with high densities. Other limitation of our pipeline is that it requires several parameters to be tuned (e.g. filter, gain, resolution). While some can be derived automatically, others cannot. A priori specification of parameter grid to explore (‘Step 2’) may be difficult, and moreover, must be repeated for each input dataset. When analysing small samples, users may need to consider several clustering methods [12] or alternative measures of impurity (e.g. using median or inter quartile range, rather than standard deviation to assess cluster impurity). Another limitation arises from a key strength of our approach: the ability to choose one or more ‘data filters’ through which to view the point cloud. While the ability to orient the clustering towards theoretically relevant variables represents a key strength, this requires users have a sense of which variables are suitable as filters, as well as the number and types to include. Finally, this procedure can be computationally expensive, especially for large datasets or when the parameter grid includes a large number of parameters.

## Conclusions

We have presented a novel pipeline built on recent advances in topological data analysis to identify homogeneous clusters with respect to a characteristic of interest. Our pipeline combines and extends existing software implementations of the Mapper algorithm to provide several unique strengths, as the integration of prior knowledge to inform the clustering process, the restriction of clusters search to significant topological features, the use of multivariable machine learning to describe clusters composition, and the ability to incorporate mixed data types.

## Availability and requirements

Project name mapper-pipeline

Project home page https://github.com/kcl-bhi/mapper-pipeline

Operating system Platform independent

Programming language Python

Other requirements Python 3.6 or higher; see requirements.txt for details.

License GNU GPLv3

Any restrictions to use by non-academics None

## Supplementary information

**Additional file 1.** Software used in the pipeline. Comparison of Mapper pipeline with k-means clustering.

**Additional file 2.** Main characteristics of the top five clusters derived with our pipeline applied to the validation dataset

## Data Availability

The data that support the findings of this study are available from the corresponding author on reasonable request. Data were used under license for the current study, and so are not publicly available.
