# Supplementary material for: Identifying homogeneous subgroups of patients and important features: a topological machine learning approach
Source: BMC Bioinformatics. 2021 Sep 20;22:449. doi: 10.1186/s12859-021-04360-9 (PMC8451168; doi:10.1186/s12859-021-04360-9)
Supplement: Supplementary file 1 — Additional file 1. Software used in the pipeline. Comparison of Mapper pipeline with k-means clustering. [file 12859_2021_4360_MOESM1_ESM.pdf]

Supplementary Materials

Software used in the pipeline

This pipeline has been implemented in Python 3 Van Rossum and Drake (2009) under a GNU GPLv3. We build on several open source packages. From *sklearn-tda* (Carrière, 2019) and *Statmapper* (Carrière, 2020) we use functions to run the Mapper algorithm, to identify representative topological features, and evaluate their significance with the bootstrap. These packages themselves build upon the GUHDI library (The GUDHI Project, 2020). We use *Graphviz* and `pygraphviz` (Hagberg *et al.*, 2020) to draw Mapper graphs and highlight topological features. We use `numpy` (Harris *et al.*, 2020) and `pandas` (The pandas development team, 2020) for data manipulation and computing statistical summaries; and XGBoost (Chen and Guestrin, 2016) to predict feature membership.

Comparison of Mapper pipeline with k-means clustering

We compared our Mapper pipeline with k-means clustering using the GENDEP dataset (Uher *et al.*, 2010). GENDEP comprises 430 individuals with major depression who were randomised to two different treatments over 12 weeks. 140 clinical, genetic, and sociodemographic variables (both categorical and continuous) were measured at baseline. Genetic variables included a polygenic risk score for depression — a score reflecting the sum of all known risk alleles for depression, weighted by how risky each variant was known to be. The outcome was a binary measure of remission at week 12 (1 = Remitted; 0 = Not remitted). Table 1 presents impurity measured by the Gini coefficient for the top five clusters derived (i) using our pipeline and (ii) using k-means clustering. The outcome was a categorical measure of remission measured at 12 weeks based on the Hamilton Rating Scale for Depression (Hamilton, 1967). Remission was defined as a score of 7 or less on the last available measure after 4-12 weeks of treatment.

We found that the top five clusters from our pipeline outperformed the five cluster solution from

k-means clustering in terms of outcome impurity. The Gini index for clusters using our pipeline ranged from 0.30 to 0.38, whilst in clusters from k-means ranged from 0.33 to 0.50. We also found that clusters from our method showed the highest reduction in impurity overall in comparison with the whole sample.

Supplementary Table 1: Impurity for binary outcome (remission at 12 weeks) based on Mapper pipeline and k-means clustering

		Gini coefficient	Percentage reduction in Gini coefficient*
Topological features from Mapper	Cluster 1	0.30	36.12
	Cluster 2	0.35	26.01
	Cluster 3	0.36	23.65
	Cluster 4	0.38	20.16
	Cluster 5	0.38	19.46
k-means clusters	Cluster 1	0.33	30.18
	Cluster 2	0.44	6.71
	Cluster 3	0.39	17.39
	Cluster 4	0.50	-5.41
	Cluster 5	0.50	-5.33

Supplementary Table 2: Key papers showing the gradual advancement of topological data analysis in recent years

Year	Method	Citation
2002	Topological persistence	Edelsbrunner *et al.* (2002); Edelsbrunner *et al.* (2000)
2005	Persistent homology	Zomorodian and Carlsson (2005)
2009	Mapper algorithm and graph	Carlsson (2009)
2017	Topological data analysis	Chazal (2016)
2018	Extended Persistent Homology	Carrière *et al.* (2018)
2020	Topological Machine Learning	Carrière and Blumberg (2020)

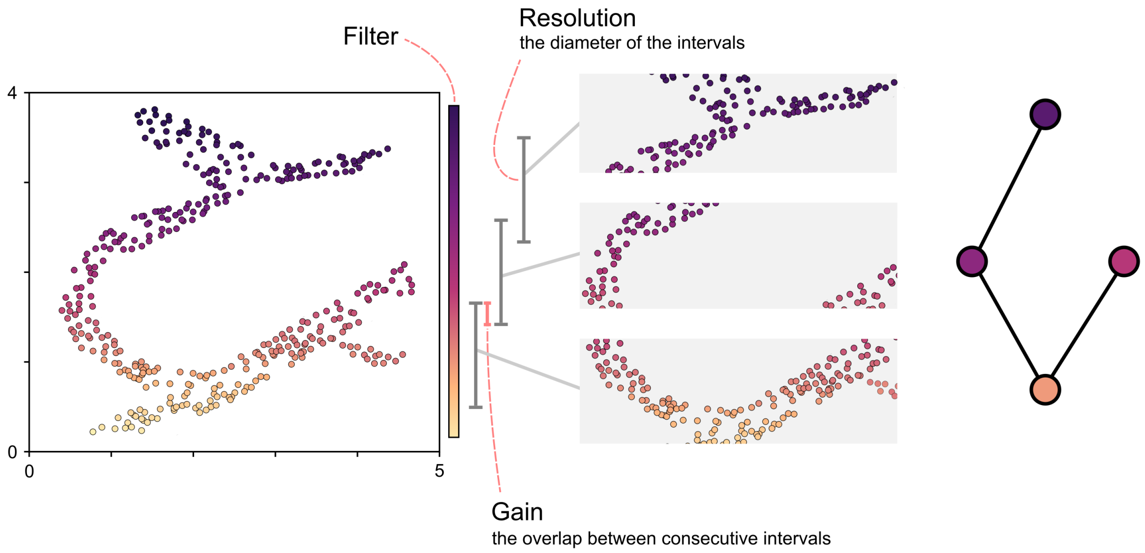

Supplementary Figure 1: Example of Mapper graph, adapted from Munch (2017). This shows the point cloud separated into intervals with diameter set by ‘resolution’ and overlap by ‘gain’.

References

Carlsson,G. (2009) Topology and data. *Bulletin of the American Mathematical Society,* **46** (2), 255–308.

Carrière,M. (2019). MathieuCarriere/sklearn-tda.

Carrière,M. (2020). MathieuCarriere/statmapper.

Carrière,M. and Blumberg,A. (2020) Multiparameter Persistence Image for Topological Machine Learning. In *Advances in Neural Information Processing Systems* vol. 33, pp. 22432–22444 Curran Associates, Inc.

Carrière,M., Michel,B. and Oudot,S. (2018) Statistical Analysis and Parameter Selection for Mapper. *Journal of Machine Learning Research,* **19** (12), 1–39.

Chazal,F. (2016) High-Dimensional Topological Data Analysis. In *3rd Handbook of Discrete and Computational Geometry.* CRC Press.

Chen,T. and Guestrin,C. (2016) XGBoost: A scalable tree boosting system. In *Proceedings of the 22nd ACM SIGKDD International Conference on Knowledge Discovery and Data Mining* KDD '16 pp. 785–794 ACM, New York, NY, USA.

Edelsbrunner, Letscher and Zomorodian (2002) Topological Persistence and Simplification. *Discrete & Computational Geometry,* **28** (4), 511–533.

Edelsbrunner,H., Letscher,D. and Zomorodian,A. (2000) Topological persistence and simplification. In *Proceedings 41st Annual Symposium on Foundations of Computer Science* pp. 454–463.

Hagberg,A., Schult,D. and Renieris,M. (2020). PyGraphviz. https://pygraphviz.github.io/.

Hamilton,M. (1967) Development of a Rating Scale for Primary Depressive Illness. *British Journal of Social and Clinical Psychology,* **6** (4), 278–296.

Harris,C.R., Millman,K.J., van der Walt,S.J., Gommers,R., Virtanen,P., Cournapeau,D., Wieser,E., Taylor,J., Berg,S., Smith,N.J., Kern,R., Picus,M., Hoyer,S., van Kerkwijk,M.H., Brett,M.,

Haldane,A., Fernández del Río,J., Wiebe,M., Peterson,P., Gérard-Marchant,P., Sheppard,K., Reddy,T., Weckesser,W., Abbasi,H., Gohlke,C. and Oliphant,T.E. (2020) Array programming with NumPy. *Nature,* **585**, 357–362.

Munch,E. (2017) A User's Guide to Topological Data Analysis. *Journal of Learning Analytics,* **4** (2), 47–61.

The GUDHI Project (2020) *GUDHI User and Reference Manual.* 3.1.1 edition,, GUDHI Editorial Board.

The pandas development team (2020). Pandas-dev/pandas: Pandas 1.1.3. Zenodo.

Uher,R., Muthén,B., Souery,D., Mors,O., Jaracz,J., Placentino,A., Petrovic,A., Zobel,A., Henigsberg,N., Rietschel,M., Aitchison,K.J., Farmer,A. and McGuffin,P. (2010) Trajectories of change in depression severity during treatment with antidepressants. *Psychological Medicine,* **40** (8), 1367–1377.

Van Rossum,G. and Drake,F.L. (2009) *Python 3 Reference Manual.* CreateSpace, Scotts Valley, CA.

Zomorodian,A. and Carlsson,G. (2005) Computing Persistent Homology. *Discrete & Computational Geometry,* **33** (2), 249–274.
